# Intracellular Redox-Modulated Pathways as Targets for Effective Approaches in the Treatment of Viral Infection

**DOI:** 10.3390/ijms22073603

**Published:** 2021-03-30

**Authors:** Alessandra Fraternale, Carolina Zara, Marta De Angelis, Lucia Nencioni, Anna Teresa Palamara, Michele Retini, Tomas Di Mambro, Mauro Magnani, Rita Crinelli

**Affiliations:** 1 Department of Biomolecular Sciences, University of Urbino Carlo Bo, Via Saffi 2, 61029 Urbino (PU), Italy; c.zara@campus.uniurb.it (C.Z.); michele.retini@uniurb.it (M.R.); tomas.dimambro@uniurb.it (T.D.M.); mauro.magnani@uniurb.it (M.M.); rita.crinelli@uniurb.it (R.C.); 2 Department of Public Health and Infectious Diseases, Laboratory Affiliated to Istituto Pasteur Italia-Fondazione Cenci Bolognetti, Sapienza University of Rome, Piazzale Aldo Moro 5, 00185 Rome, Italy; marta.deangelis@uniroma1.it (M.D.A.); lucia.nencioni@uniroma1.it (L.N.); annateresa.palamara@uniroma1.it (A.T.P.); 3 IRCCS San Raffaele Pisana, Department of Human Sciences and Promotion of the Quality of Life, San Raffaele Roma Open University, Via di Val Cannuta 247, 00166 Rome, Italy

**Keywords:** glutathione (GSH), pro-GSH molecules, redox signaling, viral infection, anti-inflammatory, antiviral

## Abstract

Host-directed therapy using drugs that target cellular pathways required for virus lifecycle or its clearance might represent an effective approach for treating infectious diseases. Changes in redox homeostasis, including intracellular glutathione (GSH) depletion, are one of the key events that favor virus replication and contribute to the pathogenesis of virus-induced disease. Redox homeostasis has an important role in maintaining an appropriate Th1/Th2 balance, which is necessary to mount an effective immune response against viral infection and to avoid excessive inflammatory responses. It is known that excessive production of reactive oxygen species (ROS) induced by viral infection activates nuclear factor (NF)-*k*B, which orchestrates the expression of viral and host genes involved in the viral replication and inflammatory response. Moreover, redox-regulated protein disulfide isomerase (PDI) chaperones have an essential role in catalyzing formation of disulfide bonds in viral proteins. This review aims at describing the role of GSH in modulating redox sensitive pathways, in particular that mediated by NF-*k*B, and PDI activity. The second part of the review discusses the effectiveness of GSH-boosting molecules as broad-spectrum antivirals acting in a multifaceted way that includes the modulation of immune and inflammatory responses.

## 1. Introduction

Most antiviral drugs target specific steps of the viral replicative cycle, i.e., adsorption and entry into the cells, reverse transcription (retroviruses), viral DNA polymerization as well as viral release and comprise inhibitors of viral entry, viral polymerase and viral proteases [1]. Nevertheless, drugs targeting viral proteins often become partially ineffective because of the rapid appearance of drug resistant strains; in fact, changes in a very small number of amino acids in the target protein can reduce the efficacy of the drug [2]. The use of such “direct” antivirals, i.e., antivirals directed against viral structures, presents some limitations, particularly in the treatment of emerging and reemerging viruses against which no vaccines or other preventive therapeutic strategies are as yet available [3].

Targeting the host cell factors required for viral infection is another therapeutic approach to fight viral infections [3]. In fact, viruses are obligate intracellular parasites depending on the host for many essential functions and exploit the synthetic machinery and energy source of the cell to ensure productive infection. Moreover, the host cells respond to the infection by activating the intrinsic defense mechanisms, which are often blocked by the virus [4]. Host-targeted antiviral therapy has emerged as a new strategy to counteract viral resistance and develop broad-spectrum antivirals [5]. The development of these new drugs is particularly urgent to treat emerging viral diseases such as Ebola, Dengue and coronavirus disease 2019 (COVID-19) for which specific treatments do not exist [3,6]. One of the most representative examples of this drug class is cyclophilin A inhibitors, which, by impairing protein folding and modulating immune responses, inhibit both RNA and DNA viruses in vitro and, as antihepatitis C virus (HCV) drugs, are in Phase II/III clinical trials [7]. Intracellular signaling pathways are therefore increasingly being studied as targets for novel antiviral therapies. Theoretically, each host factor required for a step of the viral life cycle could represent a potential target, but cytotoxicity could be a major concern [3]. Therefore, different approaches have been proposed to identify the molecular target of compounds directed to the host, from the screening of chemical libraries, genomics (i.e., gene microarrays), and/or proteomics (i.e., protein profiling) to bioinformatics approaches [8].

Endogenous thiols are of central importance in signal transduction since their redox state affects redox-modulated intracellular signaling cascades [9,10]. As many cellular redox-regulated processes are exploited by viruses to complete their lifecycle, modifications in the intracellular redox state may interfere with viral replication and be used as potential antiviral approach. Several papers have described that changes in redox homeostasis with a key feature, i.e., glutathione (GSH) depletion, favor viral replication [11,12,13,14,15]. Moreover, the efficacy of GSH and pro-GSH molecules as inhibitors of many viruses has been reported [11]. GSH can act as an antiviral by different mechanisms of action including the inhibition of the nuclear factor (NF)-*k*B signaling pathway, hindrance of the virus entry and interference with viral protein synthesis and folding [11]. It has been reported that activation of NF-kB is also required for the induction of inflammatory genes, including those encoding tumor necrosis factor (TNF)-α, interleukin (IL)-1β and IL-6 [16]. Hence, by inhibiting NF-kB-mediated signaling, GSH may also play an anti-inflammatory role and exert a protective action in inflammatory pathologies [17]. Although the addition of exogenous GSH has been found to inhibit the production of most inflammatory cytokines activated by reactive oxygen species (ROS) hyperproduction, GSH is required to restore and/or maintain interferon-γ (IFN-γ) production by antigen-presenting cells (APC), which is essential for mounting an effective immune response against intracellular pathogens [18]. GSH is essential for several functions of the immune system, both innate and adaptive, including T lymphocyte proliferation [19] and APC function [20]. Thus, the depletion of GSH may favor virus replication/propagation also by weakening the antiviral immune response. For instance, defective antigen processing and reduced IL-12 secretion correlate with GSH depletion in APC, thus favoring polarization of the Th1/Th2 response towards Th2 [21,22]. Moreover, by augmenting intracellular GSH it is possible to re-establish a balanced Th1/Th2 immune response during viral infection or to favor the Th1 immune response towards antigens [12,23,24,25]. Thus, GSH-increasing molecules may be considered broad-spectrum compounds having antiviral and immunomodulatory activity.

The first part of this review is focused on the role of intracellular GSH in: (i) redox-dependent cell signaling pathways, in particular the ones mediated by NF-kB, which can be considered the joining link between the viral replication and the cell response to contrast viral infection; (ii) redox-regulated enzymes involved in the folding and maturation of viral proteins in the endoplasmic reticulum (ER).

In the second part, some evidence about the antiviral and immunomodulatory activity of pro-GSH molecules is discussed.

## 2. Redox-Dependent Cellular Pathways as Potential Targets for New Antiviral/Anti-Inflammatory Drugs

Cellular ROS are produced intracellularly, e.g., during mitochondrial oxidative phosphorylation, or they may derive from interactions with extracellular sources such as xenobiotics. ROS contribute to both physiological and pathological conditions. Low levels of ROS are necessary for many cellular processes such as immune response, metabolism, transcriptional signaling, cell cycle, apoptosis and cell differentiation. On the other hand, when the ROS levels overwhelm the scavenging capacity of the cellular antioxidant defense system, either due to ROS overproduction or a decline in the cellular antioxidant capacity, oxidative stress and imbalance in redox equilibrium occur. Oxidative stress can damage nucleic acids, proteins, and lipids, and has been involved in several pathologies such as carcinogenesis, neurodegeneration, diabetes, and aging [26].

A strict link between energy metabolism and redox state in the immune system has been described. Metabolic pathways have an important role in immune cells because they can regulate the immune response outcome. Most of the information available concerns T cells, macrophages and dendritic cells, and in this review only some examples will be reported, but a reader interested in this topic can find more information in [27,28,29].

Immune cells have different metabolic profiles according to their function; for instance, macrophages can be distinguished into proinflammatory (M1) and anti-inflammatory (M2). M1 macrophages, responsible for inflammatory responses, utilize glycolysis to obtain energy and the pentose phosphate pathway (PPP) provides dihydro-nicotinamide-adenine-dinucleotidephosphate (NADPH) for the microbicidal pathways controlled by NADPH oxidase. Moreover, the PPP has an important role in M1 macrophages because it reduces glutathione, which then prevents the cell damage induced by ROS. On the contrary, in M2 macrophages, responsible for anti-inflammatory responses, the metabolic activity is characterized by enhanced oxidative phosphorylation.

Regarding T cells, upon antigen stimulation, these cells actively proliferate and then polarize into the different functional subsets. T cell activation and differentiation require metabolic programs that ensure adenosine triphosphate (ATP) production through oxidative phosphorylation and maintain redox balance through the generation and elimination of ROS. Increased ROS levels, derived from oxidative phosphorylation and cytosolic NADPH oxidases activity, activate redox-sensitive transcription factors that drive T cell activation and function. On the other hand, excessive ROS production is harmful and can lead to hyperinflammation and tissue damage. Excessive ROS production is regulated by GSH, which is synthesized de novo by glutamate-cysteine ligase (GCL) and glutathione synthetase (GSS). In addition, NADPH, glutathione-disulfide reductase (GSR), and glutathione peroxidase (GPX) generate GSH from glutathione disulfide (GSSG), to control oxidative stress in T cell. More details about glutathione metabolism can be found in: [30,31,32]. In conclusion, redox homeostasis is a necessary requisite for a fine-tuned T-cell mediated immune response and it derives from the coordination between the de novo synthesis of GSH and the production of ROS.

Many viruses modify the intracellular redox state toward a pro-oxidant condition, which contributes to viral pathogenesis [33]. In particular, several papers have outlined the important role of GSH, or more precisely, of the intracellular redox shift characterized by GSH depletion, in promoting an efficient virus cycle. Depending on the type of virus and the host cell, GSH decrease can occur with different kinetics and mechanisms. The most abundant data concern the alteration of GSH homeostasis in HIV infection. Both in asymptomatic human immunodeficiency virus (HIV) seropositive subjects and in HIV-infected individuals, GSH levels were found decreased in different cell types [34,35]. HIV-infected cells displayed low GSH level as well as high rate of GSSG formation [36], and T cells isolated from HIV-infected patients had lower GSH and cysteine levels [37]. Moreover, the supplementation of the GSH precursor N-acetyl-L-cysteine (NAC), as well as of the GSH ester to HIV-infected cultured monocytes, was found to inhibit HIV expression [38] and NAC administration re-established GSH blood concentrations in HIV-infected individuals [13].

The role of oxidative stress in the replication of other viruses, such as herpes simplex virus type 1 (HSV-1), Sendai and influenza viruses is well characterized both in vitro and in mouse models [39,40,41,42,43]. Besides, the administration of exogenous GSH was found to inhibit the replication of DNA and RNA viruses [11]. It has been recently hypothesized that endogenous GSH deficiency could be a critical element in amplifying severe acute respiratory syndrome coronavirus 2 (SARS-CoV-2)-induced oxidative damage of the lung and, hence, contributing to manifestations occurring in COVID-19 patients, such as acute respiratory distress syndrome, multiorgan failure, and death [44].

Many viruses modulate host redox sensitive pathways implicated in numerous cellular functions to promote viral replication and pathogenesis of the disease. The finding that GSH or pro-GSH molecules are effective in inhibiting the replication of different viruses suggests that the reducing environment created by GSH increase can inhibit the activation of oxidation-dependent signal transduction pathways associated with virus expression. Nuclear factor erythroid 2p45-related factor 2 (Nrf2) is one of the well-characterized transcription factors with an oxidants/electrophile-sensor role [45]. When normal intracellular ROS levels are present, the expression of Nrf2 is controlled by the Kelch-like ECH-associated protein 1 (Keap 1), which targets Nrf2 for ubiquitination and subsequent degradation by proteasomes. In the presence of augmented ROS production, Nrf2 escapes degradation and translocates to the nucleus, where it binds to the antioxidant-response element (ARE), with the small musculoaponeurotic fibrosarcoma (sMAF) transcription factors. Nrf2 regulates the expression of genes encoding proteins involved in cellular redox homeostasis, detoxification, macromolecular damage repair, and metabolism [45]. To control the ROS level, viruses have evolved to manipulate the Nrf2 pathway. Pathogenic viruses can regulate the Nrf2 pathway both positively and negatively [46]. This might be due to the stage of infection [47] or the specific ways through which viruses propagate; many of them kill the infected cells favoring release of virions; others survive inside the infected cells with reduction in the inflammatory response to support viral propagation [48]. For human coronavirus HCoV-229E infection, a correlation between the deficient expression of the Nrf2 target gene glucose-6-phosphate dehydrogenase (G6PDH) and increased ROS production, as well as enhanced viral gene expression, has been described [49].

Interestingly, there is functional crosstalk between Nrf2 and NF-kB in inflamed tissues, where immune cells are recruited. Indeed, among the various cellular pathways, that regulated by NF-kB offers an attractive target to viruses. After exposure to a virus, NF-kB is activated within minutes and stimulates several early viral as well as cellular genes [50]. In response to several stimuli, including viruses, the inactive cytoplasmic NF-kB/NF-kB inhibitor (IkB) complex is activated by phosphorylation: the activated enzyme IkB kinase (IKK) phosphorylates the IkBα protein, at serines 32 and 36, which results in ubiquitination, dissociation of IkBα from NF-kB and proteasome degradation of IkBα, allowing the release of NF-kB proteins. The activated NF-kB moves into the nucleus, where it stimulates the transcription of different genes. The NF-kB family encompasses five proteins (c-Rel, RelA(p65), RelB, p50/p105, and p52/p100) that share an approximately 300–amino acid NH2-terminal Rel homology domain containing sequences necessary for dimerization, DNA binding, and nuclear transport [50]. The activation of NF-kB is a hallmark of viral infections, including those caused by HIV-1, human T-lymphotropic virus (HTLV)-1, hepatitis B virus (HBV), HCV, Epstein–Barr virus (EBV), influenza virus and SARS-CoV. Although NF-kB activation is considered as a crucial factor in the innate immune response to the invading pathogen [16], it may also promote viral replication, which in turn can be impaired if the pathway is blocked. Viral products, which activate NF-kB, work by different molecular mechanisms to enhance virus replication. Here, only some examples will be reported but more information on this topic can be found in [51].

Several mechanisms have been described as regulators of NF-kB activity in HIV-1–infected cells. The fusion between HIV and the cellular membrane may lead to the activation of NF-kB via phosphoinositide 3 kinase (PI3K)/Akt signaling cascade [52]. The autocrine release of TNF-α and IL-1 may constitutively stimulate the pathways leading to NF-kB activation. The activation of the IKK complex, either directly by HIV-1 regulatory proteins or by the released cytokines, is followed by the phosphorylation and degradation of IkBα and IkBβ, thus releasing NF-kB, which translocates to the nucleus where it recognizes two consensus binding sites in the regulatory region spanning −104 to −80 in the HIV LTR, activating HIV-1 transcription. Numerous studies have shown that the activity of NF-kB can be correlated with LTR-dependent transcription and viral replication [50,51,53,54,55,56], even though other host factors, such as the nuclear factor of activated T-cells 1 (NFAT1) and NFAT5, have also been implicated in activating HIV-1 transcription through these sites [57,58]. In this regard, it has been demonstrated that low thiol levels activate NF-kB while high thiol (GSH) levels inhibit activation resulting in the regulation of the replication and expression of HIV [59].

The EBV latent membrane protein 1 (LMP1), expressed in most EBV-associated carcinomas and in Hodgkin’s lymphoma, can activate NF-kB through TNF receptor-associated factor (TRAF) 2, TNF receptor type 1-associated DEATH domain protein (TRADD) and receptor interacting protein (RIP) kinase which activate the IKK complex, by mimicking a constitutively activated TNF receptor (TNFR) [60]. HSV-1, by a series of events leading to modifications of both NF-kB and chromatin, can exploit this cellular transcription factor not only for its replication but also for its persistence within the cell [61].

Regarding the influenza virus, the expression of single proteins, as for other viruses, is sufficient to activate NF-kB through oxidative radical production and IKK activation [62]. A role for H_2_O_2_ has been clearly demonstrated in different experimental models, although other radical species have been shown to modulate the NF-kB pathway in a cell type and context specific manner [63]. Then, the different mechanisms through which NF-kB can favor influenza virus propagation have been described: it can up-regulate one or more proapoptotic factors, which result in enhanced virus production [64]; it can differentially regulate influenza virus RNA synthesis [65]; it can induce suppressor of cytokine signaling (SOCS)-3 mRNA and protein expression, which are relevant for suppression of the antiviral response [66]. Other intracellular pathways that have been found to be essential for influenza virus replication are the Raf/MEK/ERK [67] and p38MAPK [68] signaling cascades. Due to their important role for efficient influenza viral replication, these cellular pathways have been identified as promising targets for the development of novel anti-influenza drugs [69]. Indeed, several kinases involved in the cited cascades are activated by the pro-oxidant state induced by influenza virus infection, suggesting that they could be considered as useful targets for pro-GSH molecules to modulate infection [70].

**Figure 1:**
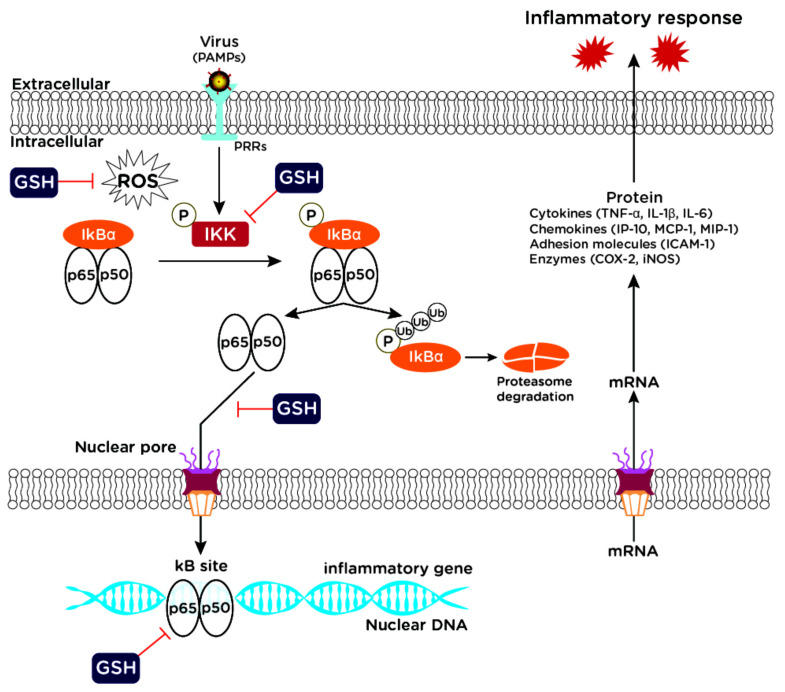
NF-kB pathway targeted by GSH. NF-kB signaling plays a role in viral infection and inflammatory response. GSH can block NF-kB signaling pathway through ROS scavenging; inhibition of IkB/NF-kB phosphorylation; interference with NF-*k*B nuclear translocation; inhibition of NF-kB binding to DNA kB site. PAMPs: pathogen-associated molecular patterns; PRRs: pattern recognition receptors; GSH: glutathione; ROS: reactive oxygen species; IKK: IkB kinase; IkBα: NF-kB inhibitor α; Ub: ubiquitin.

The signal transduction pathway modulated by NF-kB activation could be regulated by GSH at one or more points [59]. Reduced glutathione could prevent NF-kB activation simply by scavenging oxidants; it could interfere with the activation of the protein kinases that phosphorylate the IkB/NF-kB complex, thus inhibiting the release of NF-kB; it could interfere with the transport of NF-kB into the nucleus; finally, the formation of sulfenic acid and the oxidative modification of p50 could be involved in redox-induced inhibition of DNA binding to NF-kB consensus sequences (Figure 1).

On the other hand, the activation of the NF-kB transcription pathway is essential for the immediate early step of immune activation. In fact, upon viral infection, the cells initiate signaling events by pattern recognition receptors (PRRs) that recognize conserved molecular targets inside the invading pathogen proteins, referred to as pathogen-associated molecular patterns (PAMPs). The activation of the NF-kB pathway occurs when a signal molecule binds to its own receptor; some examples are given by binding of lipopolysaccharide (LPS) to Toll-like receptor (TLR) 4, the binding of cytokines to their receptors, or the recognition of RNA viruses by TLRs, i.e., TLR7/8. Remarkably, all these different signaling pathways induce phosphorylation of the cytosolic inhibitor IkBα and its following ubiquitination and proteasomal degradation resulting in release and translocation of NF-kB into the nucleus [16,71] (Figure 1).

In addition, the multimeric protein complex known as inflammasome, which triggers the release of proinflammatory cytokines IL-1β and IL-18, is induced [72]. Altogether, these effector mechanisms converge on the induction of inflammatory responses sustained by numerous cytokines and chemokines, which leads to the activation of the innate immune response and viral clearance. These cytokines induce the expression of IFN genes in the infected cell; but the proteins induced by IFNs, by a paracrine signaling, can act on nearby cells. This originates an antiviral state that serves to limit viral spreading. The antiviral function of NF-kB also explains why many viruses try to dampen or inhibit NF-kB activity by several mechanisms [71,73,74].

Although an effective antiviral immune response is necessary for viral clearance, an exaggerated response can be harmful. Proinflammatory cascades have a fundamental role in the pathogenesis of lung damage in influenza virus and SARS-CoV infections. Some cytokines, e.g., IFNs, are expected to have an antiviral effect, while the cytokine storm may contribute to pathogenesis [75] and NF-kB inhibition may represent a useful strategy to block this phenomenon. For this reason, NF-κB has been proposed as a potential therapeutic target in microbial diseases [76] and several inhibitors of the NF-kB pathway have been identified, including natural and synthetic molecules [77]. Recently, the use of NF-kB inhibitors has been also proposed as immune potentiators in next-generation prophylactic vaccines and immunotherapy applications [78]. The administration of inhibitors of NF-kB was associated with the reduced expression of proinflammatory cytokines in the lungs of mice infected with recombinant SARS-CoV-MA15 and increased mouse survival [79]. High cytokine levels, in particular IL-6, have also been described to be responsible for lung damage, inducing vascular endothelial growth factor (VEGF) expression in epithelial cells and increasing vessel permeability in SARS as well as for lethal complications of COVID-19 [80]. For this reason, therapies targeting the host immune system may be effective for COVID-19; in particular, IL-6 blockade, e.g., by inhibiting NF-kB signaling, seems to be the most promising strategy [80]. Consequently, a role for GSH in modulating IL-6 mediated cascade in COVID-19 may be hypothesized [44]. Hence, it may be postulated that on one hand GSH or GSH-replenishing molecules can be employed as antiviral drugs because of their capacity to interfere with some step of the virus life cycle and to counteract the virus-induced oxidative stress. On the other hand, by increasing the GSH level in immune cells (both T cells and macrophages), cytokine production could be specifically modulated. For example, an increase in APC GSH concentration, mainly by inducing IL-12 production, favors the Th1 response, making the host response towards the infection more effective. Nevertheless, it has been hypothesized that the same molecules could also be used as anti-inflammatory drugs, by interfering with the NF-kB-mediated pathway. A crucial aspect to be considered is that some factors, i.e., NF-kB, can regulate several components and that numerous players are involved in immune regulation; ideally, pro-GSH therapies may modulate the signaling pathways involved in the excessive inflammatory response while preserving/restoring the ability of the host cell to mount an effective antiviral response. Indeed, the exact effect on the single cytokines by pro-GSH molecules cannot be foreseen because it depends on different factors, i.e., intracellular redox state, and should be evaluated in a more complex network of mutually interacting cytokines. This aspect will also be argued below in the paragraph entitled Antiviral and Immunomodulatory Activity of Pro-GSH Molecules.

In conclusion, viral immune pathogenesis is a multifaceted process that involves a balance between the activation and inhibition of NF-kB [55]. Although NF-kB inhibition is an attractive approach for anti-inflammatory therapies, the important function of this transcription factor in the immune response and cell survival should be considered since a global inhibition of NF-kB signaling may cause severe side effects. This means that a careful analysis of all the elements leading to activation/inhibition of NF-kB in each disease is necessary for designing more specific and effective therapeutic agents.

## 3. Redox Regulation of Protein Folding as Potential Target for New Antiviral Drugs

The endoplasmic reticulum (ER) is a membranous system consisting of the nuclear envelope with an intricate network of tubules and sheets. The membrane sheets correspond to the rough ER where protein biosynthesis occurs, whereas the tubules represent the smooth ER, which possesses roles in lipid and glycogen metabolism as well as in detoxification processes. Several viruses belonging to diverse families exploit the functions of the ER to sustain different steps of their life cycle, i.e., entry, replication of the genome, assembly and release of virions [81]. Hence, ER can be considered as the central organelle in virus–host cell relationship and its components can be considered as useful targets to hit the virus. For example, polyomavirus entry is favored by disassembly of its proteins within ER [82]. Some viruses take advantage of the ER-associated protein biosynthetic machinery to translate their genome and produce structural proteins that are necessary for the formation of mature virions, e.g., the envelope glycoprotein of HIV and hemagglutinin (HA) of influenza A virus (IAV), or nonstructural proteins that are essential for the replication of the viral genome. This occurs in positive-sense RNA ((+)RNA) virus infections, such as those caused by HCV, dengue virus and SARS-CoV. Genome replication of these viruses occurs on the ER-derived membranes, which are reorganized by viral proteins creating virus replication sites [83]. Most viruses take advantage of ER functions to promote viral protein synthesis, folding and post-translational modification [84]. Consequently, in the ER of virus-infected cells the sustained production of viral proteins overloads the processing systems interfering with the folding of host proteins, leading to an increased burden of ER/Golgi trafficking. This condition induces ER stress and the subsequent activation of the unfolded protein response (UPR), a conserved cellular pathway that modulates translation, membrane biosynthesis, and the levels of ER chaperones. Interestingly, many viruses have adapted mechanisms to modulate this stress response to create a favorable environment for replication [85].

In eukaryotes, glutathione is synthesized in the cytosol and then transported to cellular compartments, including the ER where the ratio GSH/GSSG is maintained between 1:1 to 3:1 to facilitate disulfide bond formation [86,87]. Therefore, a correct ratio between GSH and GSSG in the ER lumen is necessary for efficient isomerization of S–S bonds and transformation of non-native disulfides into native ones. Protein misfolding or the inability of the protein to accomplish its proper configuration can be the consequences of alterations in the disulfide bond formation or mispairing of the cysteine residues.

The ER redox state influences the activity of protein disulfide isomerase (PDI), which has multiple roles acting as oxidoreductase and redox-regulated chaperone. PDI catalyzes the formation, isomerization and reduction of disulfide bonds and participates to the degradation of misfolded proteins through one or several CXXC cysteine-pair active-site [88,89].

The chaperone/substrate-binding activity of PDI depends on its conformation, which is strictly coupled to its redox state. PDI was defined as a redox-dependent chaperone by Tsai et al. when they discovered that PDI mediated the assembly of cholera toxin in a redox-dependent mode [90]. PDI activity is regulated by disulfide bonds-generating systems, including Ero1, the ER peroxiredoxin Prx4 and the glutathione peroxidases like enzymes Gpx7 and Gpx8. An important role of PDI has been described in some pathologies, such as cancer, neurodegenerative diseases, infertility and infectious disease [91]. Specifically, chaperones of the PDI family isomerize and reduce the disulfide bonds as well as unfolding the C-terminal arms of VP1 protein, causing the partial disassembly of polyomaviruses [82]. Above all, PDI has a fundamental role in the viral protein folding of viruses from various families (Table 1).

PDI has been described to take part to the formation of disulfide bonds between cysteines and the correct folding of HA, which mediates influenza virus entry into the host cell [92]. The essential role of PDI for the efficient oxidative folding of HA has been corroborated by the finding that treatment with PDI inhibitor decreases intramolecular disulfide bond formation and the subsequent maturation of IAV in infected lung epithelial cells, resulting in diminished viral load and proinflammatory responses [93]. A role of PDI in the life cycle of HCV and potentially of other members of the Flaviviridae family has been also described [94,95].

Among herpesvirus glycoproteins, glycoprotein g (gB) is present as a dimer in the infectious virus particles, likely linked by disulfide bridges, whose formation has been found to be affected by GSH treatment [41]. HIV-1 envelope glycoprotein also follows a complex folding process including formation of disulfide bonds in gp120, disulfide isomerization and removal of the leader sequence [96].

Accumulating evidence suggests that coronavirus proteins are subjected to various post translational modifications by the host cells [97]. For example, disulfide bond formation is required for the proper folding and trimerization of coronavirus spike (S) protein; perturbation of the redox state in the cell by addition of reducing agents affects both completed S molecules and nascent polypeptides which are reduced and unfolded [98]. Moreover, the association of the envelope (E) protein with the S protein may be mediated by the formation of disulfide bonds between the corresponding cysteines residues [99]. These findings suggest that redox state may influence both folding/maturation of single proteins as well as later assembly in coronavirus life cycle.

**Figure 2:**
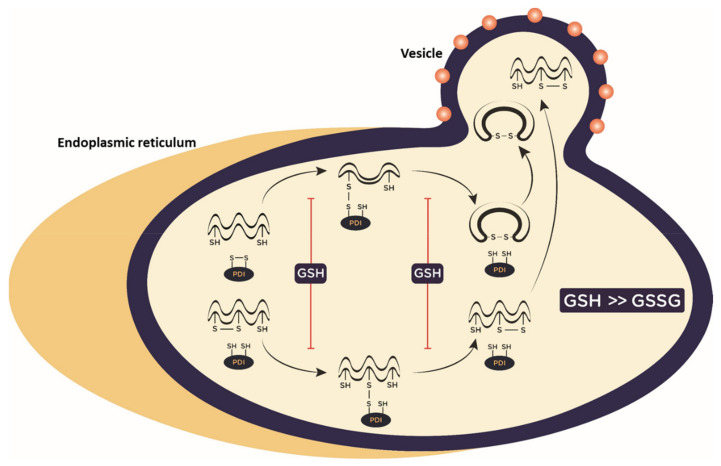
Inhibition of protein folding catalyzed by PDI through GSH in the endoplasmic reticulum (ER). PDI plays important roles in oxidative folding of proteins, catalyzing disulfide bond formation (above) or disulfide isomerization (below). GSH increase, by influencing GSH/GSSG ratio inside the ER, inhibits essential disulfide bond formation in viral proteins preventing the subsequent transport through the secretory system of the cell. GSH: reduced glutathione; GSSG: oxidized glutathione; PDI: protein disulfide isomerase.

Whether glutathione operates in the ER as a reductant or as an oxidant must be still elucidated, although there is no doubt that protein folding is susceptible to redox regulation and that GSH alterations may lead to protein unfolding/misfolding of proteins, as demonstrated by the inhibited disulfide bond formation in HA through a GSH analogue [100] (Figure 2). If viral proteins are not properly folded, viral stability and functionality, as well as immune evasion are impaired. Moreover, reduced viral release or the production of defective virions may occur.

Evidence that redox perturbation has a role in plasma cell differentiation, immunoglobulin folding and secretion has been recently given by demonstrating that in a murine model of hypergammaglobulinemia the treatment with a pro-GSH molecule induces perturbation in ER, leading to impaired IgG folding/secretion as well as to the reduced maturation of plasma cells [101]. Glutathionylation, which is the capacity of protein cysteines to form transient disulfides with GSH, is another mechanism through which the redox state of the cell can regulate protein function [102]. For example, glutathionylation of the cysteines of HIV-1 protease can abolish or stimulate enzyme activity, depending on the modified cysteine [103].

Examples of broad-spectrum antiviral drugs targeting the maturation of the viral glycoproteins by altering the ER folding machinery are represented by thiazolides, which were demonstrated to be active against IAV, hepatitis C, and paramyxovirus infection in vitro as well as in clinical studies [104,105,106,107]. Hence, redox-sensitive folding factors responsible for viral protein maturation may be considered as therapeutic targets for novel antiviral strategy.

## 4. Synthetic Molecules Able to Increase Intracellular Glutathione (GSH) Levels

**Figure 3:**
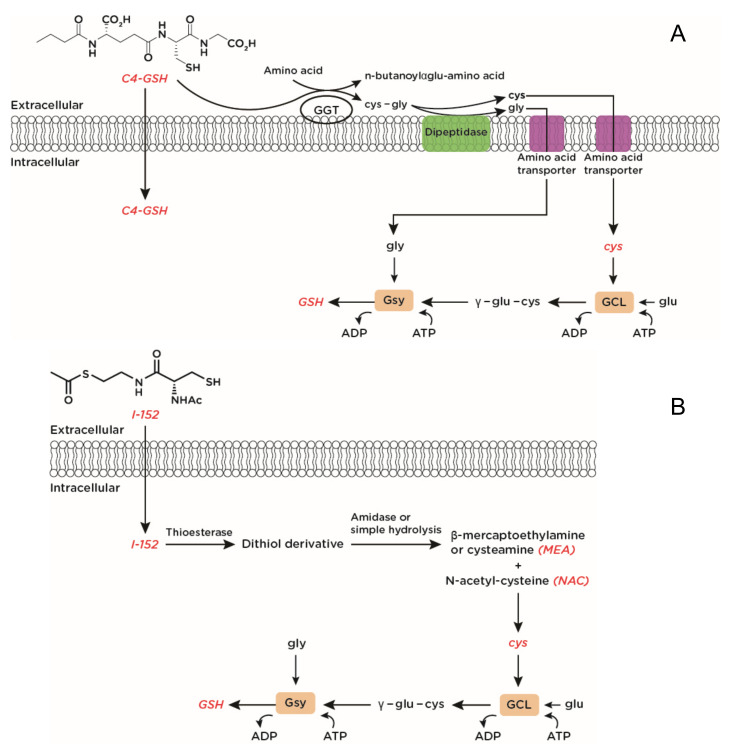
Chemical structure and metabolism of C4-GSH (**A**) and I-152 (**B**). C4-GSH, carrying a hydrophobic tail linked to the α-NH_2_ group of glutamate (glu), can go through the cell membrane. Moreover, C4-GSH can be a substrate for the enzyme gamma-glutamyl transpeptidase (γ-glutamyl transferase, GGT), which transfers the n-butanoylglutamate moiety (n-butanoylglu) to an acceptor amino acid releasing the dipeptide cysteinylglycine (cys-gly) which is further cleaved into cysteine (cys) and glycine (gly) by membrane-bound dipeptidases. These amino acids can be used to synthesize GSH inside the cell. GCL: glutamate-cysteine ligase; Gsy: GSH synthase; ADP: adenosine diphosphate; ATP: adenosine triphosphate.

Glutathione (GSH) is a tripeptide that is synthesized and maintained at high (mM) concentrations in cells [108]. The rate limiting step in GSH synthesis involves conjugation of cysteine (cys) with L-glutamate through GCL, while L-glycine is added in the consequent step by GSH synthase (Gsy) (Figure 3) [109]. Since GSH depletion is associated with a variety of diseases, including viral infections, GSH administration has been proposed to replenish the tripeptide inside the cell [110]. However, GSH’s low bioavailability and its poor capacity to cross the cell membrane limit the effectiveness of GSH as a therapeutic agent. Likewise, cys cannot be used because of its rapid oxidation to the inactive disulfide, cystine. To solve this problem, several strategies to increase GSH levels were pursued including GSH analogues, prodrug and codrug approaches. It is not the aim of this review to provide information about these strategies, but the interested reader can find details about this aspect in: [111,112,113]. We will focus on those molecules that, by increasing intracellular GSH level, influence both antiviral and immune responses.

NAC, which is the N-acetyl derivative of the natural amino acid L-cysteine, has been used therapeutically for the treatment of acetaminophen (paracetamol) overdose to replenish hepatic GSH depleted through drug conjugation [114]. NAC has also been employed as a mucolytic agent in patients with cystic fibrosis [115]. Since the 1980s, NAC has been proposed for the treatment of oxidative stress-related diseases [116]. Three different mechanisms of action have been described for NAC: a direct antioxidant effect toward some oxidant species; an indirect antioxidant effect by providing cys, which is a building block in the rate-limiting step in GSH synthesis; a reducing effect of protein disulfides through the classic thiol-disulfide interchange [117]. Recently, it has been reported that NAC can act as an antioxidant inside the cells through the conversion of NAC-derived thiols into hydropersulfides functioning as direct oxidant scavengers and/or protective caps for protein thiols [118]. Despite NAC’s therapeutic employment, many other molecules have been synthesized as an alternative to NAC with the aim to increase antioxidant potential and bioavailability of the molecule or to bypass the limits derived from the necessity that the use of NAC requires a functional enzymatic machinery for GSH synthesis. For several years, our research group has been using two GSH-boosting molecules: the n-butanoyl derivative of GSH (C4-GSH) (Figure 3A) and I-152, a codrug of NAC and cysteamine (MEA) (Figure 3B). C4-GSH and I-152 increase cellular GSH through different mechanisms. C4-GSH, as it is, can enter the cell or extracellular C4-GSH can be a substrate for the enzyme gamma-glutamyl transpeptidase (γ-glutamyl transferase, GGT) which transfers the glutamate moiety to an acceptor amino acid releasing the dipeptide cysteinylglycine (cys-gly) which is further cleaved by membrane-bound dipeptidases. The released amino acids are then taken up and used from cells for intracellular synthesis of GSH (Figure 3A). C4-GSH was synthesized with the aim to increase the lipophilicity of GSH [119]. Indeed, thanks to the addition of an aliphatic chain to the α-NH2 of glutamic acid, the hydrophobic properties of GSH are increased.

I-152, a codrug of NAC and S-acetyl-mercaptoethylamine (SMEA) linked together by an amide bond, is deacetylated to the corresponding dithiol derivative, which may release NAC and MEA (Figure 3B) [120]. It was synthesized with the aim to design a new potent lipophilic antioxidant molecule with improved delivery properties of the two compounds.

## 5. Antiviral and Immunomodulatory Activity of Pro-GSH Molecules

The antiviral activity of NAC was demonstrated against different viruses. Here, some examples will be reported. As explained above, HIV infection is characterized by GSH loss, to which several factors can contribute including inflammation, ROS production and drugs that are detoxified through GSH-consuming pathways. Furthermore, in HIV viral infection, an additional oxidative stress can derive form the production and release of the TAT protein, as demonstrated by the very low GSH levels found in TAT-transgenic mice [121,122]. The administration of NAC, by replenishing GSH levels, improves T and natural killer (NK) cell function in HIV-infected patients treated with highly active antiretroviral therapy (HAART) [123]. A concentration-dependent decrease in HIV-1 p24 protein and in reverse transcriptase activity was observed in macrophages treated with GSH or NAC [124]. NAC has also been used in the treatment of influenza virus infection [125] and of dengue-associated fulminant liver failure [126]. NAC treatment at high doses inhibited avian highly pathogenic H5N1 IAV replication and virus-induced cell death [127]. A certain protective effect of NAC was shown in animal models [128], even though recent studies questioned its anti-influenza activity limiting the efficiency to only a few viral strains [129,130]. However, it can be hypothesized that the mechanisms of action through which NAC can inhibit viral infection are mainly attributable to GSH effects, i.e., inhibition of NF-kB signaling and interference with protein folding and maturation.

I-152 antiviral activity was demonstrated in human monocyte-derived macrophages infected with HIV-1/BaL where effective doses (ED50, ED70, and ED90) of 45 ± 10 µM, 80 ± 35 µM and 195 ± 135 µM, respectively, were found [131]. There are not certain data about the mechanism of action whereby I-152 can inhibit HIV replication. However, in the light of capacity of I-152 to provide high amounts of different molecules carrying SH groups, such as NAC, MEA, cysteine and GSH [120], I-152 may interfere with both early and late steps in the HIV biological cycle.

I-152 is deacetylated to the corresponding dithiol derivative, which releases β-mercaptoethylamine, also known as cysteamine (MEA), and N-acetyl-cysteine (NAC). Cysteine (cys) provided by NAC can be used to synthesize GSH inside the cell. In red are the thiol species provided by the pro-GSH molecules.

In fact, MEA, which showed anti-HIV-1-activity both as a single agent and combined with nucleoside analogues, has been demonstrated to inhibit proviral DNA formation [132]. Moreover, NAC, by increasing intracellular GSH level, may interfere later in HIV life cycle, for example by causing a decrease in the expression of gp120, rich in disulfide bonds and therefore potentially sensitive to a more reduced environment [133].

Thereafter, I-152 was shown to be effective in reducing the infectivity of murine leukemia virus (LP-BM5) in the lymphoid organs of C57BL/6 mice, resulting in the inhibition of the main signs of the disease at a concentration 10 times lower than GSH [134,135]. Moreover, in the same animal model it was demonstrated that the disease was correlated with GSH depletion that could be prevented through I-152 treatment [12].

C4-GSH antiviral activity was demonstrated in vitro against RNA and DNA viruses, i.e., Sendai virus and HSV-1, respectively [119,136]. Moreover, C4-GSH inhibited IAV replication in cultured cells where it blocked viral HA folding and maturation by interfering with PDI activity, which is strictly dependent on the ER redox state as described above; moreover, in influenza virus-infected mice C4-GSH treatment reduced lung damage and mortality [100]. In addition, C4-GSH treatment increased GSH levels in the lymph nodes and lungs in old mice infected with mouse-adapted influenza virus (H1N1), and reduced the clinical signs of infection as well as viral copies in the lung homogenates [137].

In addition to investigating their antiviral effects, the influence of GSH-replenishing molecules on the production of macrophage inflammatory cytokines, was evaluated as well.

An inflammatory component characterizes many noninfectious diseases of the respiratory system including asthma, chronic obstructive pulmonary disease (COPD) and cystic fibrosis. Moreover, an inflammatory response has a role in the pathogenesis or complications of pulmonary infections such as tuberculosis, SARS and influenza A. Studies conducted in patients who succumb to IAV infection and in animal models show diffuse alveolar disease which may result from an excessive/unbalanced immune response or ineffective viral clearance [138,139].

The overproduction of proinflammatory cytokines (TNF-α, IL-6, and IL-1β), as mentioned above, results in what has been termed “cytokine storm”, leading to an increased risk of vascular hyperpermeability, multiorgan failure, and finally death when the high cytokine concentrations are persistent over time. In particular, respiratory viral infections (e.g., influenza A) [140] and more recently SARS-CoV-2 [141] are, in general, associated with inflammatory cytokine production, cell death, and other pathophysiological processes, which could be linked with a redox imbalance or oxidative stress which, as explained above, are crucial for viral replication and the subsequent virus-associated disease [142]. It is well established that the proinflammatory environment created by the “cytokine storm” is responsible for the lethal outcomes of COVID-19 patients [143].

It is known that respiratory viral infections induce an aberrant intracellular ROS generation resulting in an increased inflammatory cell recruitment at the site of infection [144]. Moreover, antioxidant enzymes expression is inhibited while NF-kB signaling is activated, enhancing inflammation and oxidative damage [145].

**Figure 4:**
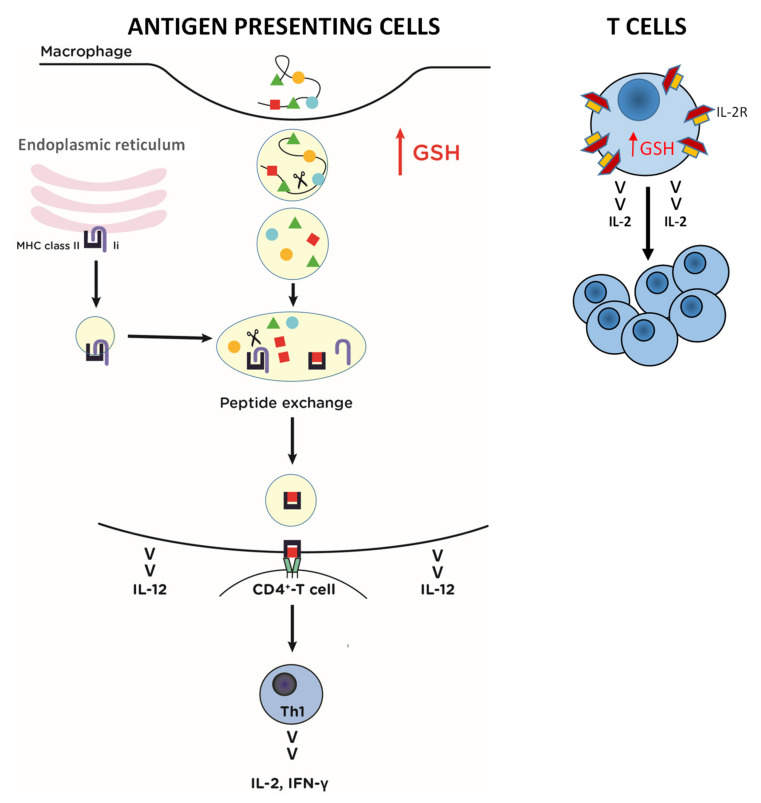
Possible effects of increased GSH in antigen presenting cells, i.e., macrophages (left) and T cells (right). In macrophages high levels of GSH favor: (1) antigen processing and presentation; (2) IL-12 production that induces differentiation of CD4^+^-Th0 cells in Th1. In T cells high levels of GSH favor: (1) IL-2 production; (2) proliferation; (3) IL-2R expression on T cell membranes. IL-12 interleukin 12; IL-2 interleukin 2; IFN-γ: interferon γ; IL-2R: IL-2 receptor; MHC II: major histocompatibility complex class II.

A balanced Th1/Th2 response is necessary for an effective and nonpathological immune response and it is profoundly influenced by the redox state of different immune cells. As reported in previous reviews [11,21], GSH can influence T cell activity by modulating the synthesis and turnover of IL-2 receptors, IL-2 production and proliferation. Moreover, in recent years increasing attention has been paid to the role of APC GSH in modulating Th1/Th2 response patterns. Most studies have been performed on macrophage cells. Briefly, macrophages with high GSH levels produce IL-12, which induces the differentiation of naive CD4+ T cells to Th1 cells and NK cells. The activated NK cells and the Th1 cells produce IFN-γ, which supports the APC’s production of IL-12. A pro-oxidant shift in the glutathione redox state during viral infection could contribute to a shift away from the typical Th1 cytokine profile towards a Th2 response compromising protection against the virus. Moreover, GSH has an important role in the reductive processing for antigen unfolding and epitope presentation, and in the maintaining of the enzymatic activity of the thiol proteases involved in the antigen processing (Figure 4).

Unbalanced/dysregulated immune response is particularly important in the elderly population whose immune system undergoes dramatic remodeling, including deficiency in the induction of Th1 cells and low-grade inflammation, referred to as “inflamm-aging”, which make the immune response ineffective against the infection [146]. Moreover, numerous studies have shown reduced vaccine efficacy in the elderly, likely due to the progressive age-related weakening of the innate and adaptive immune responses [147]. During the recent pandemic caused by SARS-CoV-2 it has become evident that aging populations are more vulnerable than young people to emerging diseases and that the development of vaccines and new immunotherapeutic agents is absolutely necessary. An accumulation of oxidative damage with aging can be considered one of the causes of the age-related decline in cellular functions and changes in redox homeostasis, in particular GSH decrease, in infected cells are linked to infection with respiratory viruses and inflammatory process [145]. For these reasons, the immunomodulatory and anti-inflammatory properties of pro-GSH molecules make them promising tools to treat old and emerging viral infections, in particular in the elderly population. A protective effect of different GSH precursors in various animal models of inflammatory pathologies of the lung has been demonstrated [17].

Regarding NAC activity, the H5N1-induced cytokine secretion was reduced by NAC in a dose-dependent manner in infected lung epithelial (A549) cells [148]. A significant inhibition of IL-8, IL-6 and TNF-α expression and secretion by NAC, likely linked to the inhibition of NF-kB nuclear translocation and phosphorylation of p38MAPK, was found in A549 alveolar type II cells infected with influenza (strains A and B) and respiratory syncytial viruses [148].

Most evidence about the regulation of inflammatory cytokines was obtained in vitro in lipopolysaccharide (LPS)-stimulated macrophage cells. Only some examples will be reported. High-dose NAC treatment was found to inhibit the expression of proinflammatory cytokines in LPS-stimulated macrophages by reducing the LPS-induced phosphorylation of Akt and IKKα/β [149]; on the contrary, low-dose NAC treatment for long periods enhanced the expression of the proinflammatory cytokines IL-1β and IL-6 in LPS-stimulated macrophages by augmenting phosphorylation of Akt and ERKs [150]. In LPS-activated THP-1 macrophages under mild oxidative conditions, high concentrations of NAC were found to inhibit the inflammatory cytokines TNF-α, IL-1β and IL-6 [151].

I-152 and C4-GSH immunomodulatory activity towards antigens was demonstrated in different animal models [24,25]. Ova-sensitized and Tat-immunized mice pretreated with I-152 or C4-GSH showed increased plasma immunoglobulin (Ig)G2a and IgG2b, characterizing the Th1 immune response, and decreased IgG1, typical of the Th2 response. Moreover, a shift to a Th1 response also involving splenocyte IFN-γ production and higher levels of IL-12 in circulation were found [24,25]. These evidences suggested that I-152 and C4-GSH can modulate the Th1/Th2 balance favoring Th1-type responses and may be proposed as Th1-directing adjuvants in new vaccination protocols and as immunomodulators in those diseases characterized by a prevalent Th2 response. Moreover, in a murine model of acquired immunodeficiency syndrome (AIDS) a correlation between GSH deficiency and a prevalent Th2 immune response was established; in the same animals, I-152 treatment was found to restore GSH content and a balanced Th1/Th2 response [12]. An immunomodulatory effect of C4-GSH treatment on the host immune response was demonstrated in aged mice infected with IAV. These animals had increased IgA in bronchoalveolar lavage fluid (BALF) as well as IgG1 and IgG2a in plasma [137]. It is known that IgG2a contribute to the clearance of the influenza virus from infected hosts since they stimulate antibody-dependent cell-mediated cytotoxicity and opsonophagocytosis by macrophages [152]. Moreover, increased IgG2a antibodies have been associated with increased efficacy of influenza virus vaccination [153], suggesting that C4-GSH may enhance influenza vaccine-specific IgG responses, particularly in the elderly subjects. Finally, C4-GSH treatment influenced the production of cytokines involved in Th1/Th2 immune response; specifically, increased IL-2 and IL-12 levels in BALF of infected old mice, driving the immune response towards the Th1 profile, were found [137].

Increased levels of IL-12, IL-2, IL-1β, and IFN-gamma were also measured in individuals with HIV infection and treated with liposomal GSH who could successfully control Mycobacterium tuberculosis infection [154].

In vitro studies aimed at elucidating the molecular mechanisms through which I-152 and C4-GSH could exert the effects observed in vivo showed that: 2h pretreatment with high dose of I-152 in peritoneal LPS/IFN-γ-stimulated macrophages blocked NF-kB nuclear translocation and prolonged signal transducer and activator of transcription (STAT)-1/ Interferon regulatory factor (IRF)-1 signaling pathway; 2h pretreatment with high dose of C4-GSH in the same experimental model favored NF-kB signaling likely by maintaining in a reduced state the highly conserved cysteine residue in the N-terminal region of the Rel homology domain [155].

On the contrary, 24-h treatment with lower dose of C4-GSH in LPS-stimulated macrophages with profound alterations in GSH-redox balance and proinflammatory cytokines production, exerted a strong anti-inflammatory effect, as revealed by inhibition of IL-1β, IL-6 and TNF-α production by interfering with NF-kB nuclear localization [156].

Hence, we can conclude that pro-GSH molecules exert opposite effects on redox-modulated cellular signaling pathways depending on different factors that will be discussed in the following paragraph.

## 6. Pro-GSH Molecules: Oxidative Stress Inhibitors or Reductive Stress Inducers?

The redox equilibrium is essential for cellular homeostasis. It is known that adequate levels of ROS act as signaling molecules involved in several cellular functions, but the excessive production of ROS, known as oxidative stress, can cause oxidative damage to cells and is often associated with ageing and many diseases [157]. As GSH depletion characterizes several pathological conditions, the use of GSH-increasing molecules has been proposed to defend the cell against the damaging effects of oxidative stress and to modulate redox-sensitive signaling as reported in this review. However, some papers have shown that molecules such as NAC can have just the opposite effect when used at high concentrations. Moreover, different effects are exerted by NAC on oxidative status in healthy animals and in animals with oxidative striatal toxicity: in healthy animals high dose of NAC increased striatal superoxide levels, and decreased GSH level; while in animals with an imbalance in redox homeostasis (a pro-oxidative state) NAC at all doses had marked protective effects against oxidative stress, suggesting that the effects of NAC were dependent on the absence or presence of oxidative stress and on the dose administered [158]. Other data indicate that NAC reduces endotoxin-related mortality thanks to its capacity to decrease oxidative stress rather than to boost GSH only when administered at low doses, otherwise, high doses of NAC increased oxidative stress and LPS toxicity [159].

Alam et al. found that in unstimulated macrophages, NAC at low concentrations (up to 3 mM) acts as an antioxidant while at higher concentrations (20 mM), it becomes a pro-oxidant [160]. The authors hypothesized that high concentrations of NAC in unstimulated cells possessing normal GSH pool create a stress which is responsible for the conversion of GSH into GSSG, transforming the cellular environment into a pro-oxidant state [160]. In agreement with these findings, Singh et al. reported that NAC could weaken cell mitochondrial respiratory chain function, leading to mitochondrial ROS production and the activation of mitochondrial biogenesis pathways [161]. Moreover, it has been demonstrated that NAC-induced apoptosis occurs via the mitochondria-dependent pathway [162]. Thus, depending on the intracellular redox state, the effects on cytokine production will be different too. In fact, a high GSH/GSSG ratio, induced by low concentrations of NAC, significantly increased IL-12 production while higher concentrations of NAC inhibited IL-12 production [163]. Apparently, contrasting results have also been obtained about the role of NAC on NF-kB activation, as it has been reported that NAC can inhibit or not LPS-induced activation of NF-kB in RAW 264.7 macrophages [164,165]. As said above, C4-GSH was found to exert different effects on NF-kB activation depending on the cellular model and the experimental conditions used [155,156] and also I-152 exerts different effects on cellular GSH pool according to the concentrations used: short treatment with high doses of I-152 (5–10 mM) induce a transient GSH depletion: on the contrary, low doses of I-152 (0.1–0.5 mM) increase intracellular GSH [120]. The exact mechanisms by which I-152 differently influences the GSH pool are under investigation but we can hypothesize that they overlap only partially with those exerted by NAC. The fact that these molecules behave differently when used at different concentrations should be considered when redox-sensitive pathways are studied. Another important factor to keep in mind is the different redox status of the cells since these molecules may specifically induce different genes depending on the cellular type and its redox status. The complex interactions of these gene products may influence the course of the mediated signaling, determining its activation or inhibition. From this point of view, the administration of pro-GSH molecules should be restricted to those physiopathologic conditions in which a redox imbalance occurs, such as in viral infections. As an alternative, an excessive amount of reducing equivalents in a cell with a balanced redox state could create what is called reductive stress, characterized by an abnormal presence in reducing equivalents, such as GSH and NADPH, increased activation of antioxidant enzymes and reduced pro-oxidant capacity, leading to a shift in the redox balance towards a reduced state [165,166]. Damage from the excessive production of ROS is a well-known phenomenon; on the other hand, reductive stress may be even more harmful than oxidative stress through several mechanisms, such as the disruption of signaling functions of ROS, the induction of ROS generation, the perturbation of cell metabolism, the inhibition of protein disulfide formation and the impairment of the proteostasis network [167]. Evidence of this is shown in a recent work where Narasimhan et al. have demonstrated that activation of the Nrf2-antioxidant signaling under basal-setting declines ROS leading to a reductive-redox state and subsequent ER stress, protein aggregation and proteotoxicity in neuroblastoma cells [168]. However, the beneficial effects of reductive stress have been also described, e.g., mitochondrial oxidation induced by NAC stimulates mitochondrial biogenesis through a mitohormesis mechanism, leading to an increased mitochondrial content and an improvement of antioxidant capacities in myoblasts [161]. Although the field of oxidative/reductive stress is extremely complex, the novel findings highlight the importance of both extremes of the redox balance, with clear evidence that oxidative and reductive stress in subcellular compartments may modulate the sensitive redox pathways often leading to disease or to the repair of pathological conditions.

## 7. Conclusions

Effective measures for the prevention and treatment of many viral infections are still lacking. Importantly, the disturbance of the host’s redox balance induced by viruses favors viral replication and impairs the immune response, triggering an excessive inflammatory response. This aspect is particularly important for respiratory virus infections where the “over” production of cytokines is often more fatal than the viruses themselves. Pro-GSH molecules are potential broad-spectrum drugs that can interfere with the protein maturation and assembly of the virus and reduce immune-inflammatory response by countering virus-induced oxidative stress and blocking redox-mediated NF-kB dependent pathways. Moreover, these molecules can induce a prevalent Th1 immune response, this aspect being particularly important for the protection of old people where an unbalance towards Th2 make these persons more susceptible to viral infections. However, important factors that should be evaluated before using pro-GSH molecules are the grade of oxidative stress triggered by the stimulus and consequently the dosage of the specific molecule to be used to increase or restore intracellular GSH level, considering the possible redox changes in all the subcellular compartments.

## Figures and Tables

**Table 1:** Viruses that exploit protein disulfide isomerase (PDI) activity to fold structural proteins.

Strain (Family)	Folded Protein	Refs
Influenza A virus (IAV) (*Orthomyxoviridae*)	Hemagglutinin (HA) glycoprotein	[92,93]
Hepatitis C virus (HCV) (*Flaviviridae*)	E1 and E2 glycoproteins	[94]
Dengue virus (*Flaviviridae*)	Pre membrane protein (preM) and E glycoproteins	[95]
Herpes simplex virus type 1 (HSV-1) (*Herpesviridae*)	Glycoprotein g (gB)	[41]
Human immunodeficiency virus (HIV) (*Retroviridae*)	gp120 glycoprotein	[96]
Coronavirus (*Coronaviridae*)	Spike (S) protein	[97,98]

## Data Availability

Not applicable.
